# Supplementary material for: Transarterial Radioembolization for Unresectable Hepatocellular Carcinoma: Real-Life Efficacy and Safety Analysis of Korean Patients
Source: Cancers (Basel). 2022 Jan 13;14(2):385. doi: 10.3390/cancers14020385 (PMC8774028; doi:10.3390/cancers14020385)
Supplement: Supplementary file 1 [file cancers-14-00385-s001.zip › cancers-1508634-supplementary.pdf]

Table S1. Treatment parameters of TARE

Variable	Patients N=149
**SIR-spheres treatment target**	
**Whole liver**	38 (25.5)
**Rt. lobe**	84 (56.4)
**Lt. lobe**	8 (5.4)
**Segment(s)**	19 (12.8)
**Whole liver volume (mL)**	1604.1 ± 600.2
**Targeted liver volume (mL)**	1097.3 ± 571.9
**Targeted tumor volume (mL)**	348.7 ± 418.4
**Tumor burden (%)**	18.1 ± 16.3
**Delivered activity (GBq)**	1.98 ± 0.89
**Tumor dose (Gy)**	235 ± 195
**Length of hospital stay**	
**Minor hospitalization (<48 h)**	24 (16.1)
**Major hospitalization (>48 h)**	125 (83.9)

Values are expressed as mean ± SD or n (%).

TARE, transarterial radioembolization.

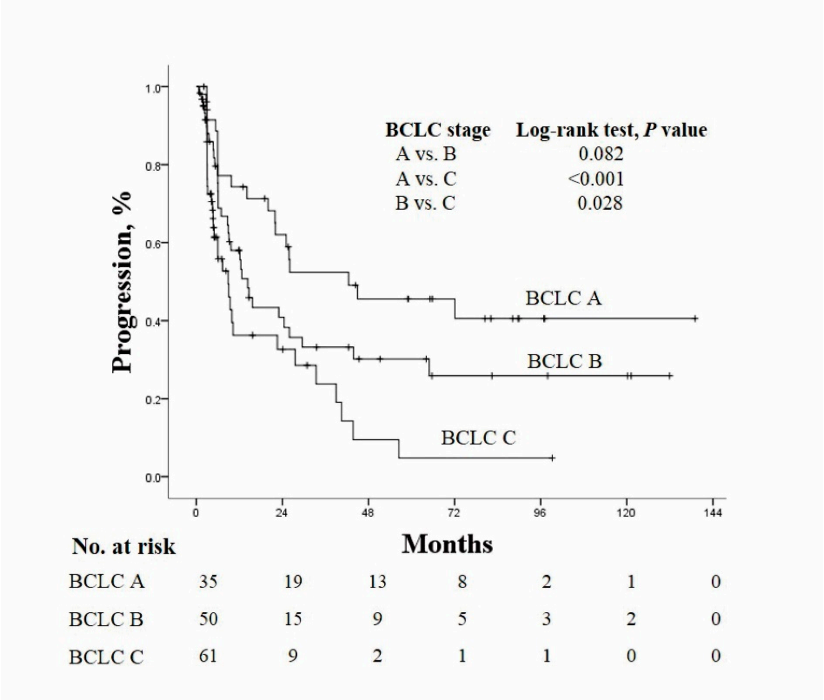

Figure S1. The Kaplan-Meier curves of progression according to BCLC stages

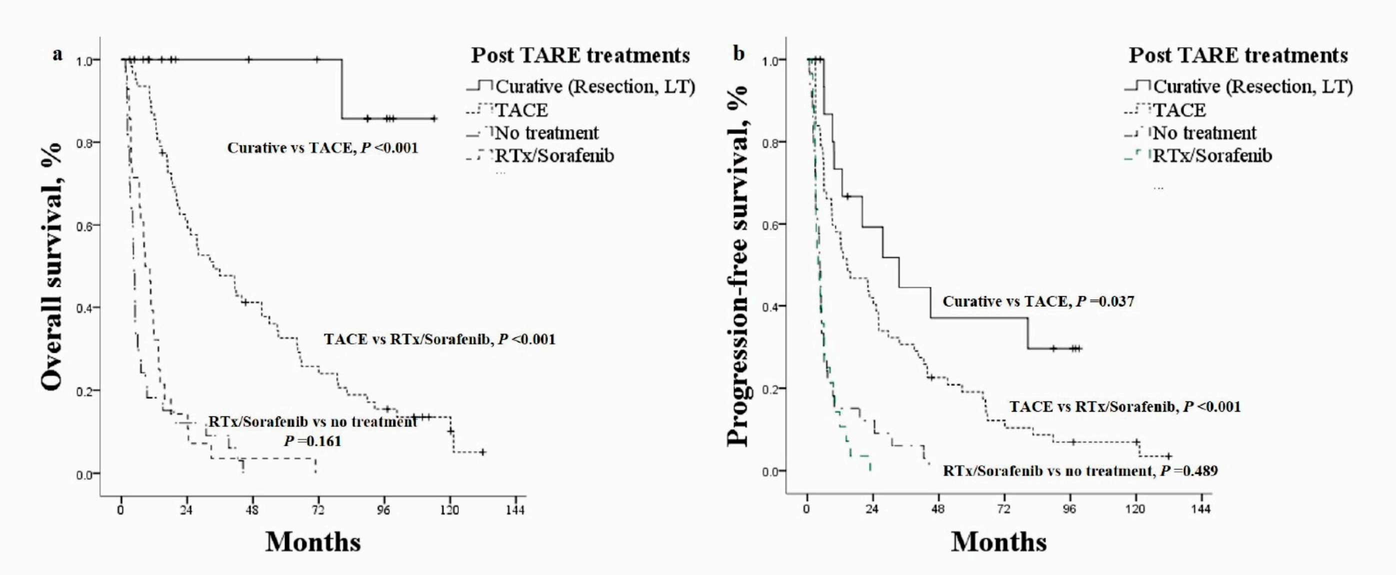

Figure S2. The Kaplan-Meier curves of (a) overall survival and (b) progression free survival according to different treatment modalities following TARE treatment
